# The development of a ternary nanocomposite for the removal of Cr(vi) ions from aqueous solutions

**DOI:** 10.1039/c9ra08298k

**Published:** 2019-11-28

**Authors:** Azza Shokry, Ayman El Tahan, Hesham Ibrahim, Moataz Soliman, Shaker Ebrahim

**Affiliations:** Department of Environmental Studies, Institute of Graduate Studies and Research, Alexandria University P.O. Box 832 163 Horreya Avenue, El-Shatby Alexandria Egypt azzashokry@alexu.edu.eg; Department of Physics, Faculty of Science, Tanta University P.O. Box 44519 Tanta Egypt; Department of Materials Science, Institute of Graduate Studies and Research, Alexandria University P.O. Box 832 Alexandria Egypt

## Abstract

The aim of this study is to develop a ternary nanocomposite (NC) of polyaniline (PANI)/2-acrylamido-2-methylpropanesulfonic acid (AMPSA)-capped silver nanoparticles (NPs)/graphene oxide quantum dots (PANI/Ag (AMPSA)/GO QDs) as an efficient adsorbent for the removal of the highly toxic hexavalent chromium (Cr(vi)) from polluted water. PANI/Ag (AMPSA)/GO QDs NC was synthesized *via in situ* oxidative polymerization. The effects of pH, adsorbent dose, initial concentration, temperature, contact time, ionic strength and co-existing ions on the removal of Cr(vi) by PANI/Ag (AMPSA)/GO QDs were investigated. The PANI/Ag (AMPSA)/GO QDs NC (25.0 mg) removed 99.9% of Cr(vi) from an aqueous solution containing 60 mg L^−1^ Cr(vi) ions at pH 2. Energy dispersive X-ray (EDX) and inductively coupled plasma spectrometry (ICP) studies confirmed the adsorption of Cr(vi) and that some of the adsorbed Cr(vi) was reduced to Cr(iii). Cr(vi) removal by the PANI/Ag (AMPSA)/GO QDs NC followed the pseudo-second order kinetic model, and the removal was highly selective for Cr(vi) in the presence of other co-existing ions. In summary, the PANI/Ag (AMPSA)/GO QDs NC has potential as a novel adsorbent for Cr(vi).

## Abstract 2

The aim is to develop a ternary nanocomposite of polyaniline/2-acrylamido-2-methylpropanesulfonic acid-capped silver nanoparticles/graphene oxide quantum dots as an efficient adsorbent for the removal of the highly toxic hexavalent chromium (Cr(vi)) from polluted water.

## Graphical abstract

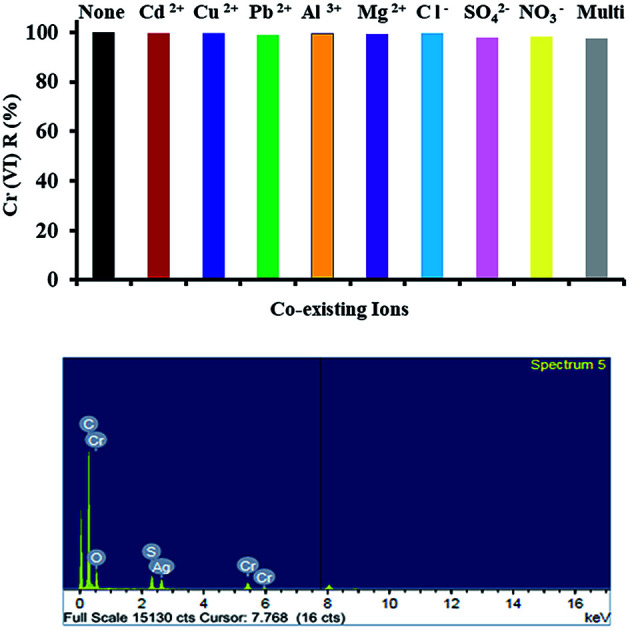

## 1. Introduction

The wastewater discharged from different industries such as the textile, tanning, electroplating, metal finishing and stainless steel production industries may contain a level of Cr(vi) higher than the permissible level. High concentrations of Cr(vi) can cause major hazards to human, animals and plants. The permissible limit of Cr(vi) for industrial effluents to be discharged to surface water is 0.1 mg L^−1^ and for potable water it is 0.05 mg L^−1^.^1^

Towards this goal, many methods and techniques have been established for removing the highly toxic Cr(vi) from wastewater and reducing Cr(vi) to the less toxic Cr(iii) prior to discharge into the environment. These methods include electrochemical methods, membrane filtration, ion exchange, chemical reduction/precipitation and adsorption.^2–8^ However, their application on a large scale faces many drawbacks such as high cost, complexity, low efficiency and sludge generation.^9^ On the other hand, owing to the simple procedure, cost-effectiveness, high efficiency, sludge free operation, potential for regeneration and environmental friendliness, the adsorption method has a great potential for the removal of Cr(vi), and thus has been attracting increasing attention in recent years.^10^

The development of new materials plays an important role by providing new characteristics for remediation/treatment applications. Graphene oxide quantum dots (GO QDs) with a particle size ranging from 2 to 20 nm are attracting considerable attention due to their superior properties such as mechanical stability, large surface area, and tunable electrical and optical properties. They are good candidates for coordinating to other molecules or materials because of important functional groups present on their surface, *i.e.* hydroxyl, carboxyl and epoxy groups.^11,12^ In addition, silver nanoparticles (Ag NPs) are anti-microbial, non-toxic, chemically stable and have a high surface-to-volume ratio. Recent advances in the synthesis of Ag NPs have been achieved through various studies related to the use of Ag NPs for the removal of heavy metals and catalysis.^13–17^

However, nanoparticles and quantum dots are prone to agglomeration, and consequently their capacity and selectivity significantly decrease. Accordingly, the most promising solution is to incorporate these materials into polymeric matrices.^18^

Polyaniline (PANI) possesses a large amount of amine and imine functional groups, which can interact with metal ions.^19–22^ PANI has been used for adsorbing different heavy metal ions due to its porous structure, regeneration, non-toxicity, insolubility in water, high stability and low cost.^19,20^ The incorporation of NPs into PANI does not only solve the agglomeration problem but can also improve the properties of the PANI matrix.^18^

Herein, we report the synthesis PANI/Ag (AMPSA)/GO QDs NC *via* an *in situ* oxidative polymerization method for the efficient removal of Cr(vi) from aqueous solution. Its removal performance for Cr(vi) was investigated using the batch technique under different environmental conditions, such as contact time, ionic strength, pH, and temperature, to explore the possible interaction mechanism.

## 2. Materials and methods

### 2.1 Materials

Aniline monomer (99.0%) was obtained from Research Lab, India. Ammonia solution 25% was received from Chem Solute, Germany. d(+)Glucose anhydrous was purchased from BDH Prolabo Chemicals. Hydrochloric acid (36.0%), sodium hydroxide (98.0%), sodium bicarbonate (99.5%) and boric acid (≥99.5%) were purchased from Sigma-Aldrich, USA. Potassium dichromate (99.0%) and sodium borohydride (99.0%) were obtained from Merck, Germany. Ammonium persulfate (APS) (98.0%), ethanol (HPLC grade), copper sulfate pentahydrate (99.0%) and magnesium sulfate heptahydrate (99.0%) were purchased from Fisher Scientific, UK. 2-Acrylamido-2-methylpropanesulfonic acid (AMPSA) (97.0%), magnesium chloride hexahydrate (98.0%) and calcium nitrate tetrahydrate (98.0%) were obtained from Acros Organics, Germany. Anhydrous lead chloride (99.0%) was received from Oxford Laboratory, India. Sodium chloride (99.0%) was received from Honeywell Company, USA. Aluminum chloride hexahydrate (95.0%) and silver nitrate (99.8%) were purchased from PRS Panreac, Spain. Dodecylbenzene sulfonic acid (DBSA) and potassium sulfate (99%) were purchased from El-Gomhoria Chemical Company, Egypt.

### 2.2 Synthesis of DBSA-doped PANI (PANI)

DBSA-doped PANI solution was prepared *via* chemical oxidative polymerization of aniline^14^ with some modifications. Aniline monomer (0.03 mL) was dissolved in 10 mL deionized water. 10 mL acidic solution of DBSA (0.3 g) and APS (0.1 g) were then slowly added over 1 h to the aniline solution with continuous stirring at room temperature until the colloidal solution turned dark green. The prepared doped PANI powder was collected by centrifugation at 7000 rpm for 8 min and washed consecutively with ethanol and deionized water. The collected PANI was dried at 60 °C.

### 2.3 Synthesis of AMPSA-capped Ag NPs (Ag (AMPSA))

Ag (AMPSA) NPs were synthesized *via* the chemical reduction of silver nitrate using sodium borohydride as a reducing agent.^23^ Freshly prepared 10 mM sodium borohydride (1.2 mL) was added to 36.8 mL of deionized water in an ice bath under continuous stirring. Then, 0.4 mL of 10 mM AgNO_3_ solution was added dropwise. The color of the solution changed to yellow, indicating the formation of Ag NPs. Finally, 0.3 mL of 10 mM AMPSA as a stabilizing agent was added dropwise to the mixture with continuous stirring for 10 min. Ag (AMPSA) NPs were separated *via* centrifugation at 8000 rpm for 10 min. The NPs were washed several times using both ethanol and deionized water. Finally, the collected Ag NPs were dried in a vacuum oven at 40 °C.

### 2.4 Synthesis of graphene oxide quantum dots (GO QDs)

GO QDs were prepared directly by glucose pyrolysis. 2 g of glucose was heated to 250 °C and after 5 min, the glucose was changed to the liquid state. The color of this liquid changed from colorless to orange within 20 min. This orange liquid was added dropwise to 100 mL of 12.5% ammonia solution under vigorous stirring. Then the solution was heated at 70 °C for 3 h until the odor of ammonia vanished and the pH was neutralized to 7. The volume of the GO QDs solution was maintained at 50 mL. The GO QD powder was separated by heating and evaporation of the GO QD solution at 200 °C for about 2 h.

### 2.5 Synthesis of PANI/AMPSA-capped Ag (PANI/Ag (AMPSA)) NC

PANI/Ag (AMPSA) NC was prepared *via* the *in situ* oxidative polymerization of aniline in the presence of Ag (AMPSA) NPs. Aniline monomer (0.03 mL) was dissolved in 10 mL of previously prepared Ag (AMPSA) NPs. 10 mL acidic solution of DBSA (0.3 g) and APS (0.1 g) were then slowly added to the aniline solution with continuous stirring at room temperature until the colloidal solution turned dark green. The prepared PANI/Ag (AMPSA) NC powder was collected by centrifugation at 7000 rpm for 8 min and washed consecutively with ethanol and deionized water. The collected NC was dried in a vacuum oven at 40 °C.

### 2.6 Synthesis of PANI/GO QDs NC

The nanocomposite of PANI/GO QDs was prepared *via* the *in situ* oxidative polymerization of aniline in the presence of GO QDs. 0.03 mL aniline monomer was dissolved in 9 mL deionized water. 1 mL of the previously prepared GO QDs solution was added under vigorous stirring at room temperature for 10 min. Then, 10 mL solution of DBSA (0.3 g) and APS (0.1 g) was slowly added to the aniline and GO QDs solution with continuous stirring at room temperature until a dark green color was observed. The green powder of PANI/GO QDs NC was collected through centrifugation and washing with ethanol and deionized water, then dried in a vacuum oven at 40 °C.

### 2.7 Synthesis of PANI/AMPSA-capped Ag/GO QDs (PANI/Ag (AMPSA)/GO QDs) NC

The PANI/Ag (AMPSA)/GO QDs NC was prepared *via* the *in situ* oxidative polymerization of aniline in the presence of freshly prepared Ag (AMPSA) NPs and GO QDs.^23^ The ternary NC was prepared by mixing 10 mL of AMPSA-capped Ag NPs and 1 mL of the previously prepared GO QDs solution under magnetic stirring for 10 min. Aniline monomer (0.03 mL) was added to the above mixture under continuous stirring for 10 min. 10 mL of DBSA (0.3 g) and APS (0.1 g) aqueous solution was added dropwise with stirring at room temperature until the dark green colored nanocomposite colloid was obtained. The prepared PANI/Ag (AMPSA)/GO QDs NC powder was collected by centrifugation at 7000 rpm for 8 min and washing with ethanol then deionized water. The collected NC was dried in a vacuum oven at 40 °C.

### 2.8 Characterization techniques

The prepared Ag (AMPSA) NPs, GO QDs and the ternary nanocomposite were characterized *via* UV-vis and photoluminescence spectroscopy to study their optical properties, their structural and morphological properties were investigated using Fourier transform infrared spectroscopy, Raman spectroscopy, X-ray diffractometry and high-resolution transmission electron microscopy (HRTEM), and the particle size distribution and average particle size of the Ag (AMPSA) NPs were determined using a particle size analyzer. The results were introduced in our recently accepted article.^23^ Elemental analysis of PANI/Ag (AMPSA)/GO QDs NC before and after treatment of Cr(vi) was performed using HRTEM coupled with energy dispersive X-ray (EDX) (JEOL, JEM-2100 LaB6). Samples were prepared by dispersing 2 mg of powder in 5 mL of ethanol. A drop of these colloidal solutions was evaporated on a copper grid and tested. The surface charges of PANI/Ag (AMPSA)/GO QDs NC at different pH values were measured *via* zeta potential measurement (Zetasizer Nano-ZS). Suspensions were placed in a universal folded capillary cell equipped with a platinum electrode. The zeta potential values were calculated from the mean electrophoretic mobility, as determined by laser doppler anemometry (LDA).

### 2.9 Cr(vi) removal measurements

#### 2.9.1 Equilibrium studies

To investigate the Cr(vi) removal, a stock solution of 1000 mg L^−1^ Cr(vi) was prepared by dissolving potassium dichromate (2.835 g) in 1000 mL of deionized water. The desired concentrations of Cr(vi) solutions were obtained by diluting the appropriate amount of Cr(vi) stock solution in deionized water. Batch experiments were carried out by mixing 25 mg (equivalent to 1 g L^−1^) of PANI/Ag (AMPSA)/GO QDs NC and 60 mg L^−1^ in 25 mL of Cr(vi) aqueous solutions in 100 mL conical flasks. These mixtures were stirred at a speed of 200 rpm at 30 °C for 60 min. The nanocomposite adsorbent was separated from the solutions by filtration using filter paper and the clear filtrates were analyzed for residual Cr(vi) concentration by 1,5-diphenylcarbazide using a UV-visible spectrophotometer (Evolution 300, Thermo scientific, USA) operated at a wavelength of 540 nm.^24^

The effects of various parameters such as pH, adsorbent dose, initial Cr(vi) ion concentration, contact time, temperature, ionic strength and co-existing ions on the Cr(vi) removal from aqueous solution using PANI/Ag (AMPSA)/GO QDs NC were investigated. The Cr(vi) removal using GO QDs, Ag (AMPSA) NPs and PANI was also studied. In addition, the removal of Cr(vi) from two local water samples using PANI/Ag (AMPSA)/GO QDs NC was carried out. The initial pH of the Cr(vi) solution was varied and adjusted from 2 to 12 using HCl or NaOH solution (0.1–1 M) to investigate the effect of pH on Cr(vi) adsorption by the adsorbent. For the effect of adsorbent dose, the mass of the PANI/Ag (AMPSA)/GO QDs NC was varied from 0.2 to 1.6 g L^−1^. For the effect of initial Cr(vi) ion concentration, the concentration of Cr(vi) ions was varied from 0.01 to 200.00 mg L^−1^. The effect of temperature and adsorption process was investigated at five different temperatures with a Cr(vi) concentration of 60 mg L^−1^. The effect of ionic strength on the removal of Cr(vi) was studied by adding NaCl with concentrations ranging from 0 to 1000 mM to 60 mg L^−1^ Cr(vi) solution. The effect of selected co-existing heavy metal cations including Cd(ii), Cu(ii), Pb(ii), Al(iii) and Mg(ii) in solution with a concentration of 60 mg L^−1^ on the removal of Cr(vi) using PANI/Ag (AMPSA)/GO QDs NC was studied. Furthermore, the effect of these mixed cations (where each cation has a concentration of 60 or 100 mg L^−1^) on the removal of Cr(vi) using the prepared nanocomposite was also investigated. In addition, three common coexisting salts, NaCl, K_2_SO_4_ and Ca(NO_3_)_2_ with a concentration of 60 mg L^−1^ for each salt were used to study the effect of Cl^−^, NO_3_^−^ and SO_4_^2−^ anions on the removal of Cr(vi).

The removal percentage (*R*, %) and the removal capacity (*q*, mg g^−1^) of Cr(vi) were calculated using the following equations:^25^1
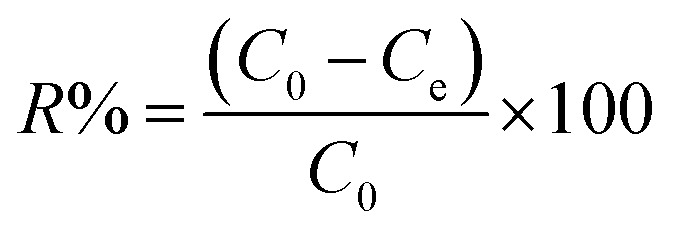
where *C*_0_ and *C*_e_ are the initial and final metal ion concentration, respectively, in (mg L^−1^).2
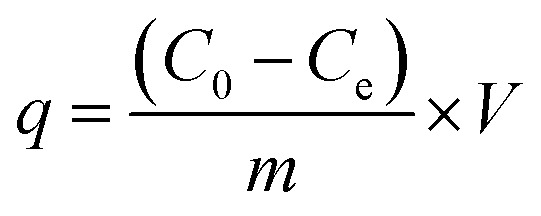
where *q* is the amount of Cr(vi) ions adsorbed per unit mass of the adsorbent (mg g^−1^), *V* the volume of sample (L) and *m* is the adsorbent dosage (g).

#### 2.9.2 Kinetic studies

The kinetics of the removal of Cr(vi) was studied for three initial Cr(vi) concentrations (10, 30 and 60 mg L^−1^) to investigate the effect of contact time ranging from 10 to 70 min on Cr(vi) adsorption by the PANI/Ag (AMPSA)/GO QD NC by adding 100 mg adsorbent to 100 mL (1 g L^−1^) of Cr(vi) solution at pH 2 and 30 °C with stirring. 10 mL of Cr(vi) solution was withdrawn at different times for Cr(vi) analysis. For the kinetic studies, the capacity of the adsorbent, *q*_*t*_, at time *t* was obtained using eqn (3):^25^3
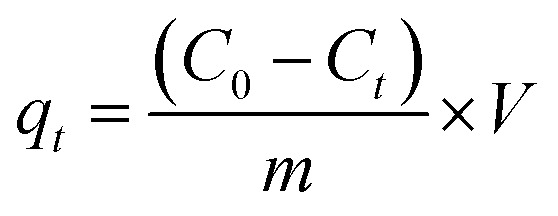
where *q*_*t*_ is the amount of Cr(vi) ions adsorbed per unit mass of the adsorbent (mg g^−1^) at time *t* and *C*_*t*_ (mg L^−1^) is the Cr(vi) concentration at time *t*.

#### 2.9.3 The removal of Cr(vi) from water samples

The application of the nanocomposite for the removal of Cr(vi) from two water samples (tap water and raw water obtained from a drinking water canal, Alexandria, Egypt) was applied. The raw water was filtered to remove impurities. The two water samples were analyzed by applying the colorimetric method using 1,5-diphenylcarbazide (DPC). No detectable Cr(vi) ions were found in the two water samples and consequently the water samples were spiked with a fixed Cr(vi) concentration (60 mg L^−1^) and the removal of Cr(vi) ions by mixing 25 mL of each water sample with 1 g L^−1^ of PANI/Ag (AMPSA)/GO QDs NC at pH 2 for 60 min was tested.

#### 2.9.4 Regeneration and reusage of PANI/Ag (AMPSA)/GO QDs NC

An ideal adsorbent should not only display high removal efficiency, but also possess an excellent regeneration and recycling performance, which is important for water remediation. Therefore, the regenerability and reusability of PANI/Ag (AMPSA)/GO QDs NC after Cr(vi) adsorption were investigated by adsorption–desorption experiments. The desorption of 60 mg L^−1^ Cr(vi) from 25 mg (1 g L^−1^) PANI/Ag (AMPSA)/GO QDs NC was carried out using 0.5 M NaOH solution. After desorption, the PANI/Ag (AMPSA)/GO QDs NC was treated with 2 M HCl to desorb the Cr(vi) ions, which were reduced to Cr(iii), and regenerate the adsorbent.^25^ The desorption efficiency (%) was calculated using the following eqn (4):^26^4
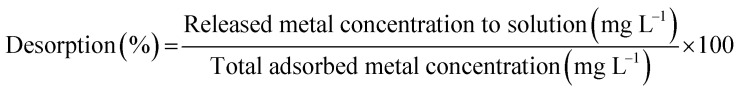


After washing with deionized water until the pH was approximately 7, PANI/Ag (AMPSA)/GO QDs NC was dried in a vacuum oven for further use as an adsorbent for the removal of 60 mg L^−1^ Cr(vi) from aqueous solution. Three consecutive adsorption–desorption cycles were carried out at pH 2.

## 3. Results and discussion

### 3.1 Cr(vi) removal using PANI/Ag (AMPSA)/GO QDs NC

#### 3.1.1 Effect of pH

**Fig. 1:**
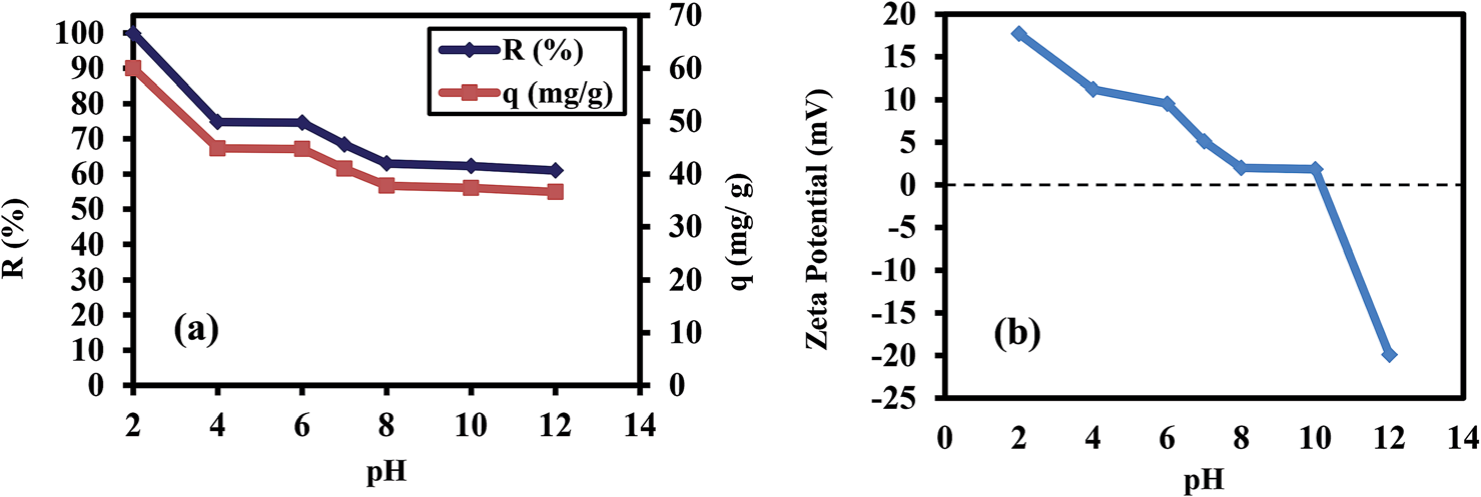
(a) *R*% and *q vs.* pH for 25 mL Cr(vi) solution (60 mg L^−1^) mixed with 1 g L^−1^ PANI/Ag (AMPSA)/GO QDs NC after 60 min at 30 °C and (b) zeta potential of PANI/Ag (AMPSA)/GO QDs NC *vs.* pH.

The pH dependence of the Cr(vi) removal percentage (*R*) and removal capacity (*q*) was studied in the pH range of 2 to 12 with 60 mg L^−1^ Cr(vi) and 25 mg (1 g L^−1^) of PANI/Ag (AMPSA)/GO QDs NC after 60 min at 30 °C, as shown in Fig. 1a. The Cr(vi) *R* and *q* decreased from ∼100% to 61.0% and from 59.96 mg g^−1^ to 36.61 mg g^−1^, respectively, as the solution pH increased from 2 to 12, where the maximum *R* and *q* was found at pH 2. From acidic pH 1 to neutral pH 7, the hydrogen chromate (HCrO_4_^−^) ions are the dominant ions, whereas above neutral pH, only chromate ions (CrO_4_^2−^) are present in the solution.^27^ The maximum adsorption observed at pH 2 is attributed to the increase in the positive charges on the adsorbent surface, resulting in electrostatic attraction between the positively charged adsorbent and the negative HCrO_4_^−^ ions.^27^ Also, the nitrogen atoms in PANI were protonated and attracted Cr(vi) anions, which replaced the –SO_3_^−^ dopants ions of the PANI/Ag (AMPSA)/GO QDs NC through the ion-exchange process.^10,28,29^

With an increase in the pH of the solution, HCrO_4_^−^ was gradually converted to other ion forms such as CrO_4_^2−^ and Cr_2_O_7_^2−^. At a higher pH value, the competition between the negatively charged OH^−^ ions in the adsorption medium and CrO_4_^2−^ species for the adsorbent sites caused a decrease in the efficiency of the adsorbent for the removal of Cr(vi) ions.

To understand how electrostatic forces affect the Cr(vi) adsorption behavior under different pH values, the relation between the zeta potential of the PANI/Ag (AMPSA)/GO QDs NC and pH from 2 to 12 was investigated, as shown in Fig. 1b. The zeta potentials confirm the presence of surface charges on the PANI/Ag (AMPSA)/GO QDs, which decrease sharply with an increase in the pH value. It was observed that the isoelectric point of PANI/Ag (AMPSA)/GO QDs NC is at about pH 10.3. The maximum positive and negative charges of +17.7 and −19.9 mV were found at pH of 2 and 12, respectively. Therefore, at higher pH, the repulsion between anionic chromium spices with negative surface charges on the PANI/Ag (AMPSA)/GO QDs nanocomposite plays a major role in decreasing the adsorption efficiency.^10,25,29–32^ Although, the *R* of Cr(vi) from solution at pH 12 using PANI/Ag (AMPSA)/GO QDs NC is only 61%, this value is much higher than other adsorbents.^28–30,33^

#### 3.1.2 Mechanism of Cr(vi) removal

The proposed mechanism involved in the removal of Cr(vi) on the adsorbent can be understood as follows. The enhancement in the adsorption at lower pH is governed by three mechanisms: (i) electrostatic interaction, (ii) anion exchange and (iii) reduction process of Cr(vi) to Cr(iii).^10,28–30,34^ From the viewpoint of electrostatic interaction, a lower pH is suitable for the adsorption of anionic Cr(vi). Meanwhile, the polyaniline layer can be partially converted from half-oxidized emeraldine form into its fully oxidized pernigraniline state after treatment with Cr(vi). This behavior is shown in eqn (5):^35^

5 2HCrO_4_^−^ + 14H^+^ + 3PANI^2+^ → 2Cr^3+^ + 3PANI^4+^ + 8H_2_O

where PANI^2+^ refers to the “half-oxidized” emeraldine form of PANI doped with DBSA and PANI^4+^ represents PANI in the fully oxidized pernigraniline structure. However, the reduced Cr(iii) is released into the solution due to the electrostatic repulsion between the positive charge of the Cr(iii) ions and the positive charges existing on the surface of the nanocomposites. Another portion of the converted Cr(iii) species may be chelated on the amine groups on the PANI/Ag (AMPSA)/GO QDs surface. This is based on the hypothesis that the nitrogen atoms in polyaniline related to the PANI/Ag (AMPSA)/GO QDs NC can form coordinate bonds with the positively charged Cr(iii) due to the presence of a lone pair of electrons on nitrogen.^19,35,36^ In addition, the removal of Cr(vi) species in alkaline solution (pH 12), where CrO_4_^2−^ is the dominant ion in solution, may be attributed to the precipitation of Cr(iii) hydroxide.^35^

**Fig. 2:**
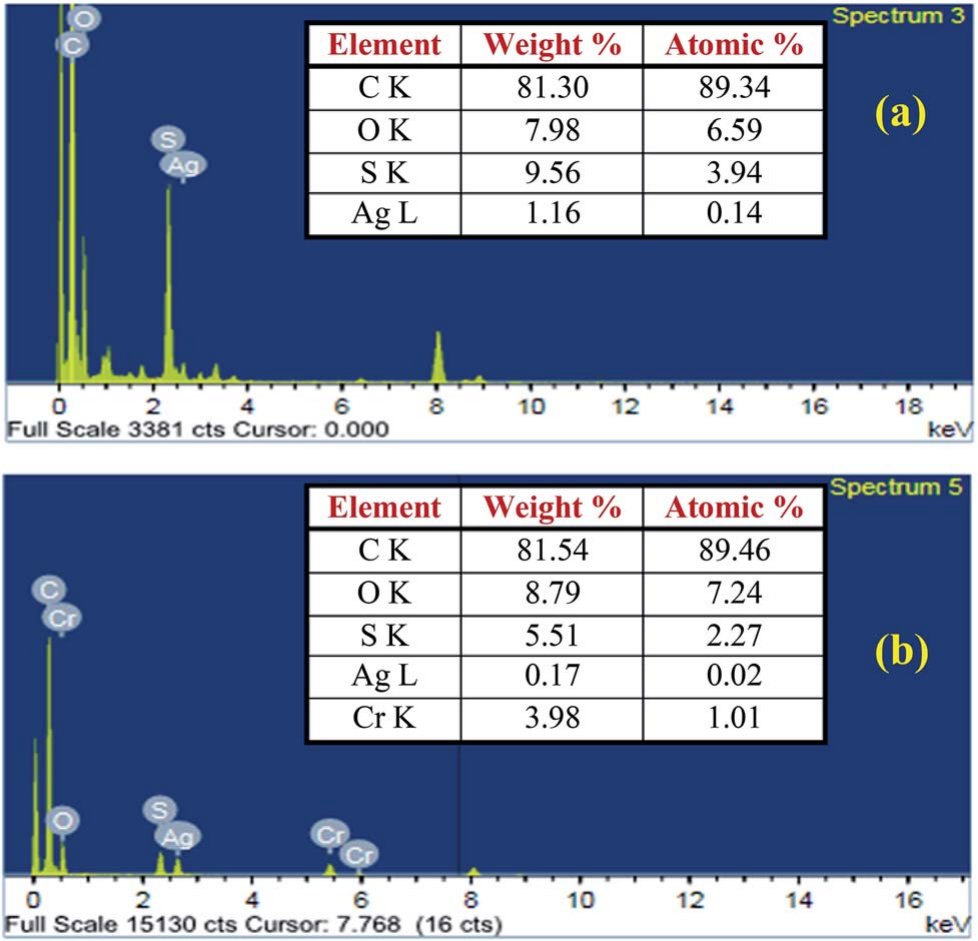
EDX analysis of PANI/Ag (AMPSA)/GO QDs NC (a) before and (b) after treatment with Cr(vi).

The EDX analysis was used to investigate the mechanism of Cr(vi) removal from aqueous solution at pH 2 by the PANI/Ag (AMPSA)/GO QDs NC. The EDX spectra obtained for PANI/Ag (AMPSA)/GO QD NC before and after Cr(vi) adsorption are shown in Fig. 2a and b, respectively. The EDX studies of PANI/Ag (AMPSA)/GO QDs NC showed a peak for silver. The presence of an Ag peak strongly indicates that Ag (AMPSA) NPs were successfully decorated on the PANI or PANI layer-coated Ag (AMPSA) NPs. Moreover, no Cr ions signals were observed in the EDX spectrum of PANI/Ag (AMPSA)/GO QDs before Cr(vi) adsorption (Fig. 2a). However, the EDX spectrum of the chromium-loaded PANI/Ag (AMPSA)/GO QDs NC indicates the adsorption of Cr(vi) ions, as shown in Fig. 2b. According to the EDX results, the chromium content on the NC surface increased from 0 to 1.01 atomic%, while the S content was reduced after Cr(vi) adsorption from 3.94 to 2.27 atomic%. There is no doubt that this relative content of S and Cr is related to the release of S and the adsorption of Cr(vi) *via* the anion exchange process between Cr(vi) and –SO_3_^−^ dopant ions on the NC surface during Cr(vi) adsorption, respectively.

The reduction of Cr(vi) to Cr(iii) was confirmed by analyzing the total Cr and Cr(vi) ions in solution with pH 2 containing 60 mg L^−1^ Cr(vi) after the adsorption process with 1 g L^−1^ of PANI/Ag (AMPSA)/GO QDs NC using ICP (CIROS VISION, Germany) and UV-vis spectrophotometry. The concentrations of total Cr and Cr(vi) ions were found to be 29.80 mg L^−1^ and 0.04 mg L^−1^, respectively. Subsequently, the estimated concentration of Cr(iii) ions was determined to be 29.76 mg L^−1^. The adsorbed amount of Cr(vi) is about 30.24 mg L^−1^. These results confirm the proposed mechanism of the removal process of Cr(vi) by the PANI/Ag (AMPSA)/GO QDs NC.

#### 3.1.3 The effect of adsorbent dose

**Fig. 3:**
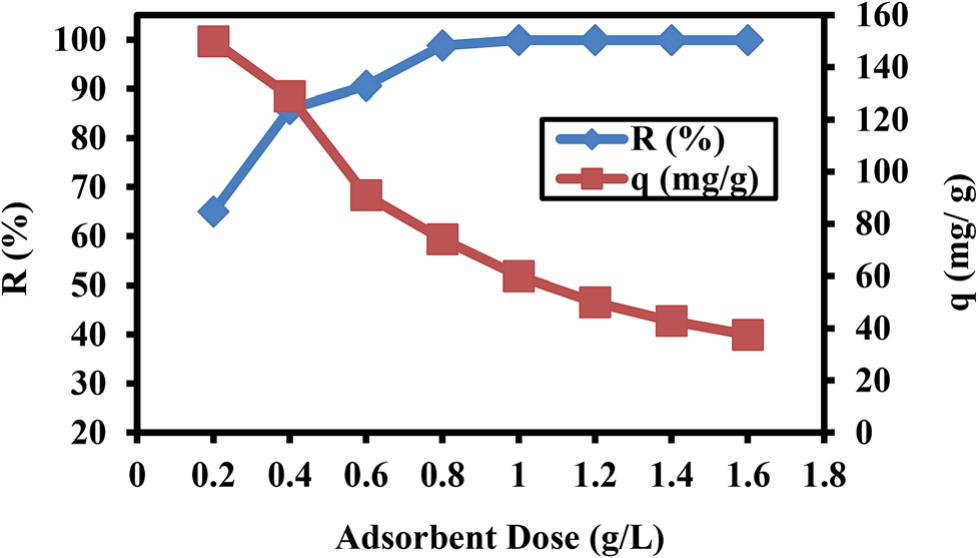
*R*% and *q vs.* adsorbent dose for 25 mL Cr(vi) solution (60 mg L^−1^) at pH 2 after 60 min and at 30 °C.

Optimization of the adsorbent dosage for the removal Cr(vi) ions is crucial to determine the minimum amount of absorbent required for obtaining the maximum adsorption. The experiments were conducted by introducing different adsorbent dosages (0.2 to 1.6 g L^−1^) to 25 mL of 60 mg L^−1^ Cr(vi) solution at pH 2 for 60 min at 30 °C. The results obtained from Fig. 3 show that increasing the adsorbent dosage from 0.2 g L^−1^ to 1.0 g L^−1^ led to an enhancement in the removal of Cr(vi) from 65.0% to 99.9%. This is mainly due to the increase in the number of active sites available for the adsorption Cr(vi) ions on PANI/Ag (AMPSA)/GO QDs NC.^32,33,37^ The efficiency of the PANI/Ag (AMPSA)/GO QDs NC with 1 g L^−1^ for Cr(vi) removal was 99.9% after 60 min, and the residual concentration of Cr(vi) was 0.0370 mg L^−1^, which meets the standards for the Cr(vi) level in surface water (0.05 mg L^−1^) set by the WHO.^1^ Therefore, 1 g L^−1^ of adsorbent is sufficient for the for the quantitative removal of chromium from wastewater with an initial Cr(vi) concentration of 60 mg L^−1^. In addition, there is a plateau region for the removal of Cr(vi) with an adsorbent dose higher than 1 g L^−1^ due to all the Cr(vi) ions in the solution being removed. On the other hand, the *q* of Cr(vi) shown in Fig. 3 decreased from 194.95 mg g^−1^ to 37.48 mg g^−1^ with an increase in the PANI/Ag (AMPSA)/GO QDs NC dose from 0.2 to 1.6 g L^−1^, which can be attributed to the formation of aggregates that reduce the availability of the effective sorption area. Similar results are reported in other research works.^19,38,39^

#### 3.1.4 The effect of initial Cr(vi) ion concentration

**Fig. 4:**
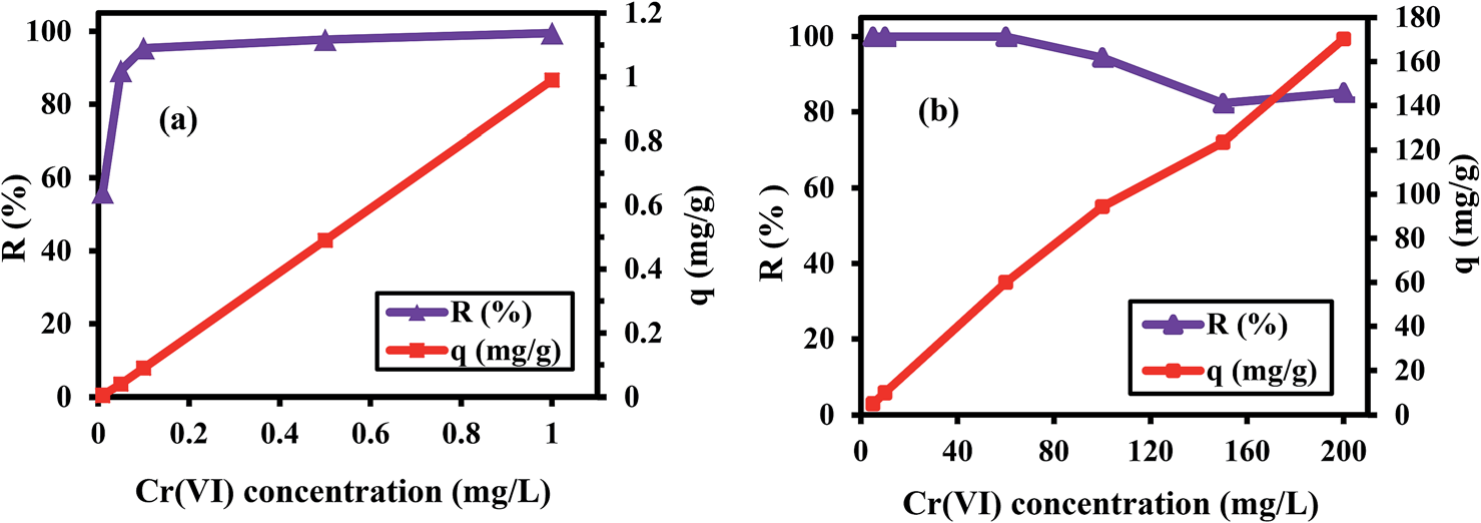
*R*% and *q vs.* Cr(vi) concentration ranging from 0.01 to 1 mg L^−1^ (a) and from 5 to 200 mg L^−1^ (b) at pH 2 and 30 °C after 60 min.

The role of the concentration of Cr(vi) ions on the removal process was studied by changing the concentration of Cr(vi) from 0.01 to 200 mg L^−1^ at pH 2 for 60 min, as illustrated in Fig. 4. By increasing the Cr(vi) ion concentration from 0.01 to 0.1 mg L^−1^, the *R* suddenly increased and attained a maximum value of about 99.9% at 1 mg L^−1^, as displayed in Fig. 4a. This is may be due to the availability of the adsorption sites, which initially leads to a sharp increase in the Cr(vi) adsorption.^19,40^ The decline in *R* from 99.9% to 85.1% at higher concentrations of Cr(vi) ranging from 60 to 200 mg L^−1^, as shown in Fig. 4b, is because the available binding sites on the surface of the PANI/Ag (AMPSA)/GO QDs NC are saturated and limited. The excess ions remain in the solution when the Cr(vi) ion concentration in the solution exceeds a certain value of 100 mg L^−1^.^37^ The over oxidation and degradation of polyaniline can be also increase in the oxidative environment of a high concentration of Cr(vi) ions.^35,41^

The q values shown in Fig. 4a and b are found to linearly increase from 0.01 to 170.23 mg g^−1^ with an increase in the concentration of Cr(vi) from 0.01 to 200 mg L^−1^. Increasing the concentration of Cr(vi) provides a driving force to overcome the mass transfer resistance of the metal ions between the aqueous and solid phases, and this results in a higher probability of collision between the Cr(vi) ions and sorbent.^42–44^

#### 3.1.5 Adsorption kinetics

**Fig. 5:**
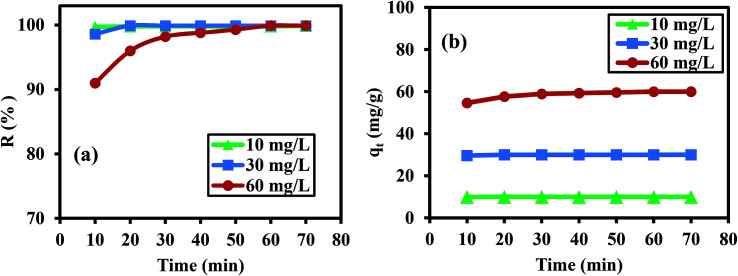
*R* (a) and *q*_*t*_ (b) *vs.* contact time for different concentrations of Cr(vi) ions on 1 g L^−1^ PANI/Ag (AMPSA)/GO QDs NC at pH 2 and 30 °C.

The effect of contact time on the Cr(vi) adsorption on the PANI/Ag (AMPSA)/GO QDs NC surface was studied for three different Cr(vi) concentrations (10, 30 and 60 mg L^−1^) at pH 2 and 30 °C, as depicted in Fig. 5a. At about 10 min of contact time, high *R* values of 99.7%, 98.6% and 91.0% were obtained for 10, 30 and 60 mg L^−1^ Cr(vi), respectively. It was observed that for 60 mg L^−1^ Cr(vi), the adsorption of Cr(vi) ions increased with an increase in contact time until equilibrium was attained at 60 min due to the availability of abundant active sites on the nanocomposite surface.^19^ On the other hand, for 10 and 30 mg L^−1^ Cr(vi), the *R* values were high at small contact time of 10 min and remained fixed with an increase in the contact time. This suggests that the adsorption process is faster at lower initial concentrations due to the higher degree of freedom for the distribution of the adsorbate ions on the surface of the adsorbent.^30^ Thus, it can be concluded that the optimum times for the removal of Cr(vi) using PANI/Ag (AMPSA)/GO QDs NC are 10, 20, and 60 min for 10, 30 and 60 mg L^−1^ Cr(vi), respectively.

Fig. 5b displays the relationship between *q*_*t*_ and contact time. Similarly, *q*_*t*_ rapidly increased to a high value in a short time for 60 mg L^−1^ Cr(vi) and achieved 54.61 mg g^−1^ at 10 min. Then, it slightly increased with time until the adsorption capacity of 59.97 mg g^−1^ was obtained at 60 min. The rapid Cr(vi) adsorption can be attributed to the abundant adsorption sites on the surface of the adsorbent and the significant concentration drop between the liquid and solid phases in the initial stage, which led to the easy capture of Cr(vi) by the adsorbent in the solution. As the adsorption sites became covered and the concentration difference declined, it became difficult for Cr(vi) to capture the remaining sorption sites. This result is attributed to the repellency between the Cr(vi) ions in the solid and liquid phases, resulting in a lower adsorption rate until equilibrium is reached.^32,45^ The highest capacities (*q*_e_) for 10 and 30 mg L^−1^ Cr(vi) of 9.99 and 29.98 mg L^−1^ were achieved at 10 and 20 min, respectively.

The mechanism of Cr(vi) adsorption on the PANI/Ag (AMPSA)/GO QDs NC was investigated using the pseudo-first and pseudo-second order models. The linear form of the pseudo-first order kinetic model (Lagergren, 1898)^46^ is shown in eqn (6):6
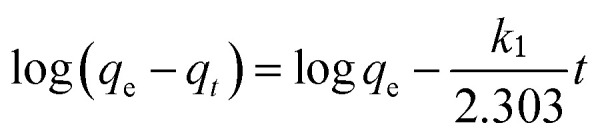
where *q*_e_ (mg g^−1^) and *q*_*t*_ (mg g^−1^) are the adsorption capacities at equilibrium and time *t* (min), respectively, and *k*_1_ (min^−1^) is the pseudo-first order rate constant of adsorption.

By plotting log(*q*_e_ − *q*_*t*_) *versus t*, the constant *k*_1_ (min^−1^) and *q*_e_ (mg g^−1^) were determined from the slope and intercept of the obtained line, respectively.

The pseudo-second-order model (Ho and McKay, 1998)^47^ assumes that the adsorption of an adsorbate onto the adsorbent supports second order chemisorption. The pseudo-second order equation is represented with eqn (7):^47^7
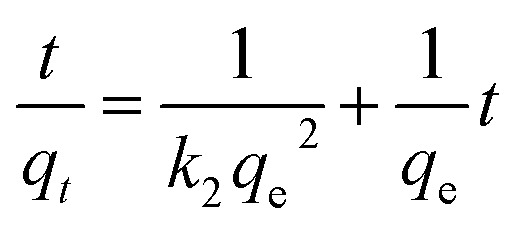
where *k*_2_ (g mg^−1^ min^−1^) is the pseudo-second order rate constant of adsorption. By plotting *t*/*q*_t_*versus t*, the constant *k*_2_ (g mg^−1^ min^−1^) and *q*_e_ (mg g^−1^) are determined from the intercept and slope of the straight line, respectively.

**Fig. 6:**
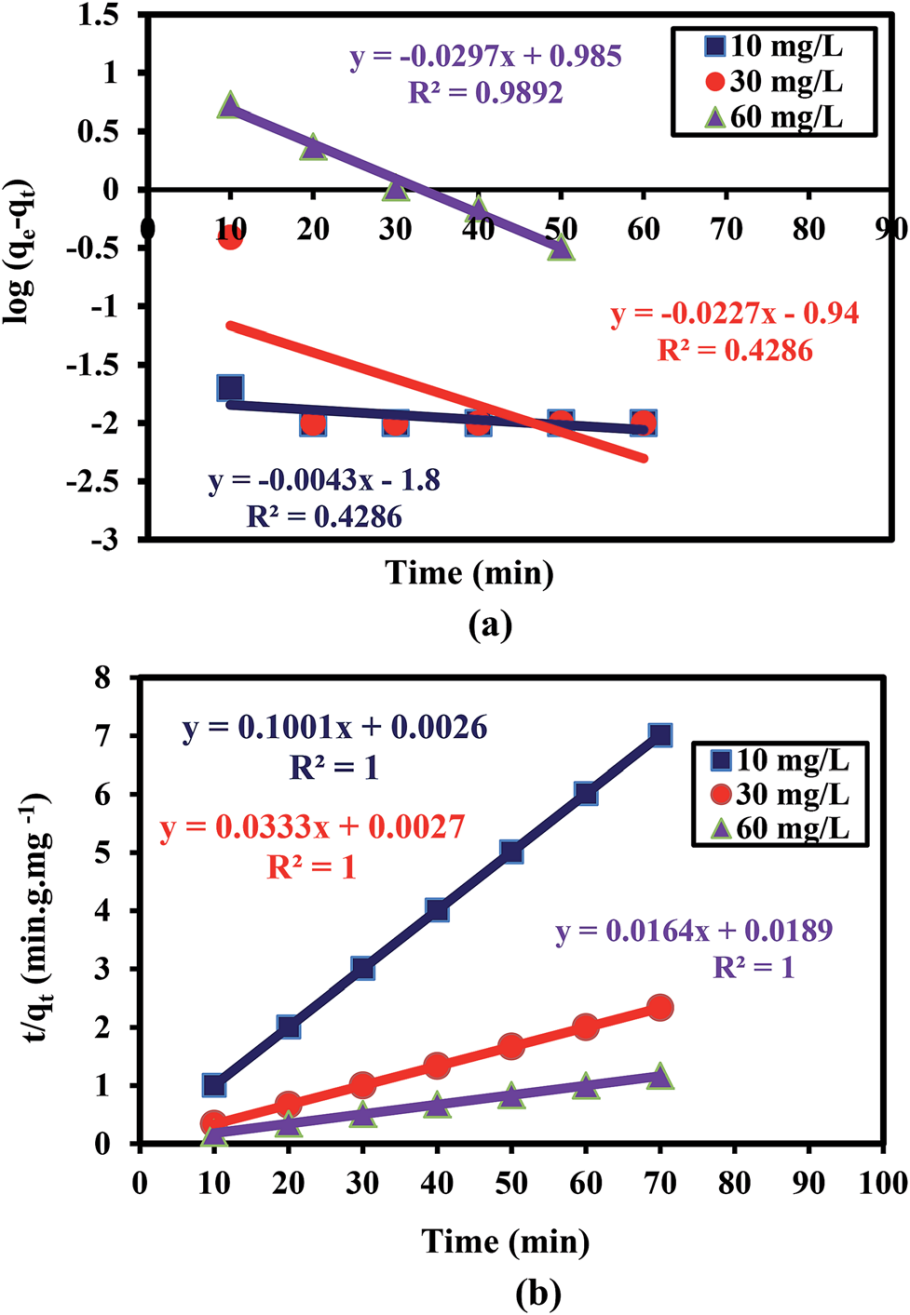
(a) Pseudo-first order and (b) pseudo-second order kinetic model plots of the adsorption of different concentrations of Cr(vi) ions on the PANI/Ag (AMPSA)/GO QDs NC at pH 2 and 30 °C.

Fig. 6a and b show the kinetic data fitting to the pseudo-first order and pseudo-second order kinetic models, respectively. Table 1 presents the rate constants and other parameters obtained from the linear regression. For the pseudo-first order kinetics fitting, the determination coefficient (*R*^2^) values are less than 1 for the 10 and 30 mg L^−1^ Cr(vi) solutions, and the estimated *q*_e_ values of 0.02, 0.11 and 9.66 mg g^−1^ are less than that observed from the experiments (9.99, 29.98, and 59.97 mg g^−1^) for 10, 30 and 60 mg L^−1^ Cr(vi) solution, respectively. This indicates that Cr(vi) adsorption on the PANI/Ag (AMPSA)/GO QDs NC does not follow pseudo-first order kinetics. On the other hand, it was found that the *R*^2^ values obtained by applying the pseudo-second order model are about 1. The estimated *q*_e_ values are consistent with that obtained experimentally in the case of the pseudo-second order model (9.99, 30.03 and 60.97 mg g^−1^) in 10, 30 and 60 mg L^−1^ Cr(vi) solution, respectively. The *k*_2_ values decreased with an increase in the Cr(vi) concentration, suggesting that rate of Cr(vi) adsorption on PANI/Ag (AMPSA)/GO QDs NC increased as the Cr(vi) concentration decreased.^33^ These results confirm that Cr(vi) adsorption by the PANI/Ag (AMPSA)/GO QDs NC follows pseudo-second order kinetics and chemisorption (chemical interaction such as complexation and redox reaction) occurs.^47^

**Table 1:** Kinetic parameters of pseudo-first order and pseudo-second order models for the adsorption of different concentrations of Cr(vi) on the PANI/Ag (AMPSA)/GO QDs NC

Kinetic model	Initial Cr(vi) concentration		
	10 mg L^−1^	30 mg L^−1^	60 mg L^−1^
**Pseudo-first order**			
*q*_e_ (mg g^−1^)	0.02	0.11	9.66
*k*_1_ (min^−1^)	0.01	0.05	0.07
*R* ^2^	0.4286	0.4286	0.9892

**Pseudo-second order**			
*q*_e_ (mg g^−1^)	9.99	30.03	60.97
*k*_2_ (g mg^−1^ min^−1^)	3.85	0.41	0.01
*R* ^2^	1	1	1

#### 3.1.6 The effect of the temperature

**Fig. 7:**
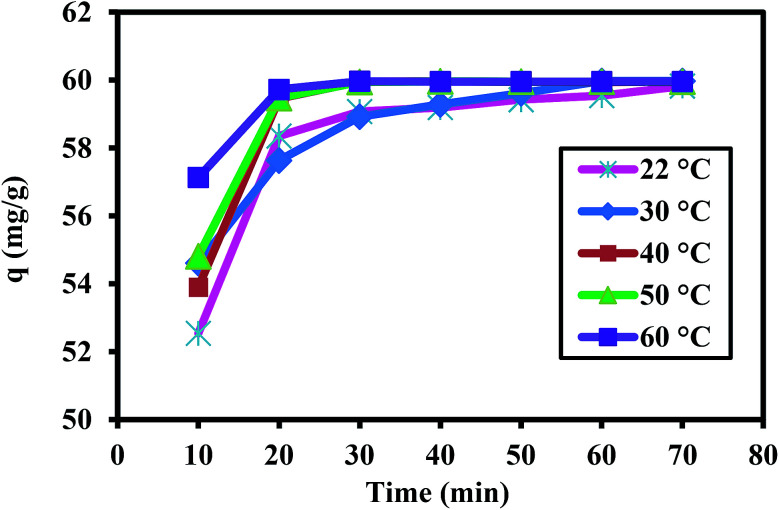
*q vs.* time for Cr(vi) concentration of 60 mg L^−1^ at pH 2 at different temperatures.

**Fig. 8:**
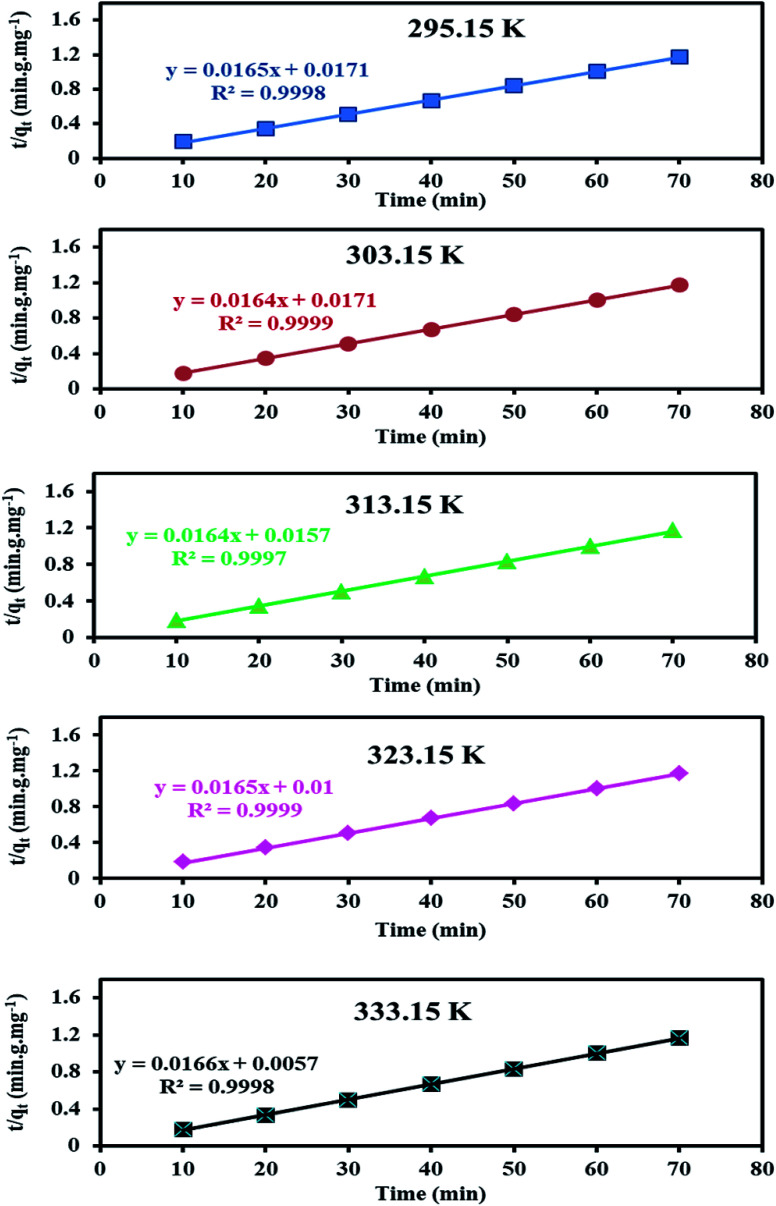
Pseudo-second order kinetic model plots for the adsorption of 60 mg L^−1^ Cr(vi) on the PANI/Ag (AMPSA)/GO QDs NC at different temperatures and pH 2.

The effect of temperature on the removal capacity of 60 mg L^−1^ Cr(vi) ions by 1 g L^−1^ PANI/Ag (AMPSA)/GO QDs NC was studied at pH 2 for time intervals ranging from 10 to 70 min, as shown in Fig. 7. The kinetic experiments show that the rate of Cr(vi) adsorption on the surface of PANI/Ag (AMPSA)/GO QDs NC increased as the temperature increased from 22 °C to 60 °C, which suggests that adsorption process is endothermic and spontaneous.^29,33,45,48^ The enlargement of pore size and activation of the adsorbent surface at high temperature promote rate of adsorption.^45,49^ The rate constant *k*_2_ for each temperature (Table 2) was obtained by fitting the data to the linear pseudo-second order kinetic model, as shown in Fig. 8.

**Table 2:** Pseudo-second order kinetic parameters for the adsorption of 60 mg L^−1^ Cr(vi) on PANI/Ag (AMPSA)/GO QDs at different temperatures

*T* (°C)	*T* (K)	1/*T* (K^−1^)	*k*_2_ (g mg^−1^ min^−1^)	ln *k*_2_ (mg L^−1^ min^−1^)
22	295.15	0.0034	0.010	−4.6
30	303.15	0.0033	0.016	−4.6
40	313.15	0.0032	0.017	−4.0
50	323.15	0.0031	0.033	−3.4
60	333.15	0.0030	0.049	−3.0

**Fig. 9:**
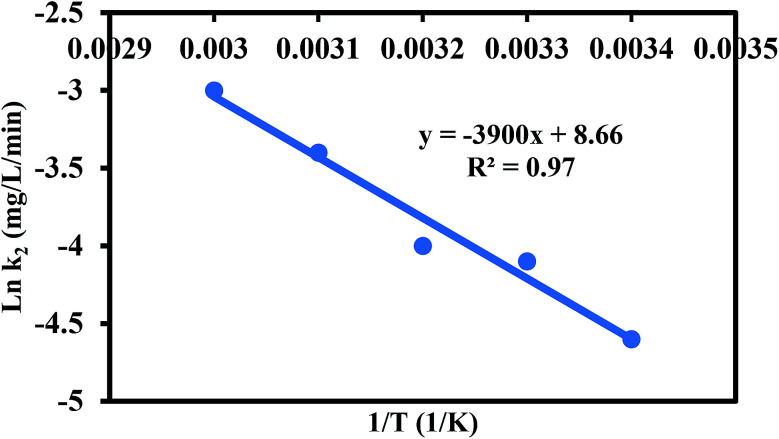
Plot of ln *k*_2_*vs.* 1/*T* for the adsorption of Cr(vi) on PANI/Ag (AMPSA)/GO QDs NC.

By applying Arrhenius eqn (8),^33^ the activation energy (*E*_a_, kJ mol^−1^) for the adsorption of Cr(vi) on the surface of the PANI/Ag (AMPSA)/GO QDs NC was obtained from the slope of the plot of ln *k*_2_ against 1/*T* (K^−1^) (Fig. 9).8
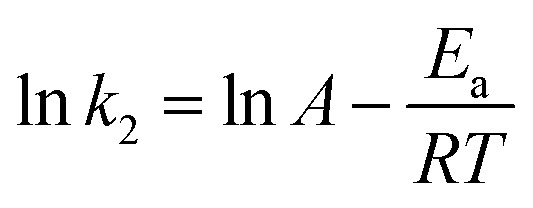
where *k*_2_ (g mg^−1^ min^−1^) is the pseudo-second order rate constant at a certain temperature (*T*), *A* is the pre-exponential factor (g mg^−1^ min^−1^) and *R* is the ideal gas constant (0.008314 kJ mol^−1^ K^−1^).

*E*_a_ was estimated to be 32.4 kJ mol^−1^, suggesting the chemisorption mechanism occurred. Activated chemisorption means that the rate of removal varies with temperature according to a finite activation energy (between 8.4 and 83.7 kJ mol^−1^) in the Arrhenius equation.^30,50^

#### 3.1.7 The effect of ionic strength and co-existing ions on Cr(vi) removal

**Fig. 10:**
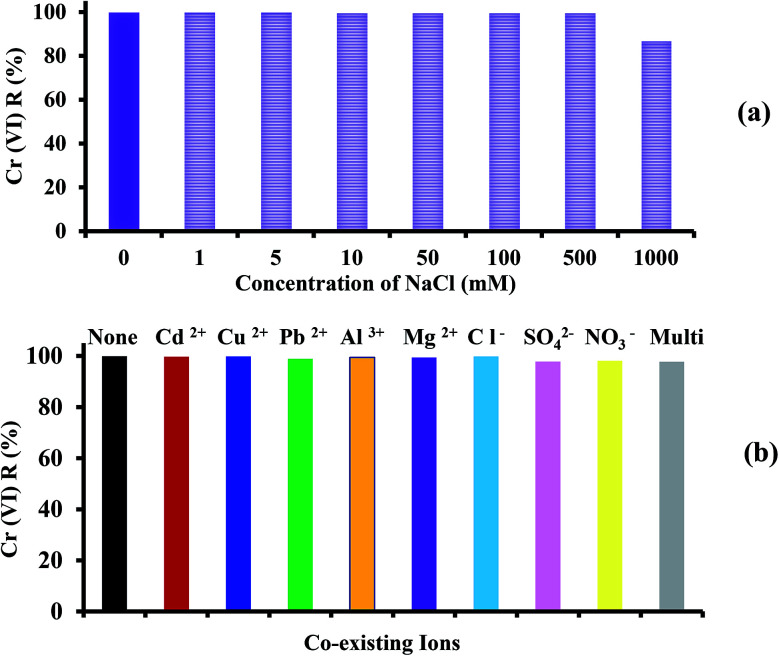
Effect of (a) ionic strength and (b) co-existing ions on the removal of 60 mg L^−1^ Cr(vi) by 1 g L^−1^ PANI/Ag (AMPSA)/GO QDs NC at pH 2 and 30 °C after 60 min.

To investigate the effect of ionic strength on the removal of 60 mg L^−1^ Cr(vi), different concentrations of NaCl ranging from 0 to 1000 mM were mixed with the Cr(vi) solution at pH 2, 30 °C and adsorbent dose of 1 g L^−1^ for 60 min, and the results are presented in Fig. 10a. The competitive Cl^−^ had no effect on the Cr(vi) adsorption and the *R* of Cr(vi) remained fixed. This is attributed to the weak competition from the monovalent Cl^−^ ions and the strong covalent bonds between the Cr(vi) ions and amine groups of PANI.^45^ In addition, a further increase in NaCl concentration to 1000 mM led to a decline in *R* by 12.8% due to the reduction in the number of collisions between Cr(vi) ions and PANI/Ag (AMPSA)/GO QDs.^48^

Industrial wastewater also contains other types of heavy metal cations and anions such as Cd(ii), Cu(ii), Pb(ii), Al(iii) and Mg(ii). Therefore, it is essential to investigate the competitive influence of these co-existing ions on the Cr(vi) removal percentage using the PANI/Ag (AMPSA)/GO QDs nanocomposite. Initially, the Cr(vi) removal was recorded using 25 mL of 60 mg L^−1^ of Cr(vi) solution with 25 mg (1 g L^−1^) of PANI/Ag (AMPSA)/GO QDs NC. The effect of metal cations with individual concentrations of 60 mg L^−1^ on the Cr(vi) removal was studied, as shown in Fig. 10b. It was noted that the presence of 60 mg L^−1^ initial concentration of individual cations in solution did not affect the removal of Cr(vi). These cations ions are repelled from the positively charged surface of the PANI/Ag (AMPSA)/GO QDs NC.^12^ In addition, Cr(vi) adsorption occurs through anion exchange with doped –SO_3_^−^ ions, and therefore the nanocomposite has little or no affinity for the cation ions.^33^ Other anions salts may also compete with the removal of Cr(vi) ions on the PANI/Ag (AMPSA)/GO QDs NC available binding sites. In addition, three common coexisting salts, NaCl, K_2_SO_4_ and Ca(NO_3_)_2_, were chosen to study the effect of their anions on the removal of Cr(vi). The results are shown in Fig. 10b. NaCl, Ca(NO_3_)_2_ and K_2_SO_4_ barely influenced the removal of Cr(vi). The reason for this is that Cl^−^ and NO_3_^−^ are monovalent anions, and they would slightly compete for the adsorption sites of PANI/Ag (AMPSA)/GO QDs NC.^51^ Besides, these anions have low affinity ligands, which mostly form weak outer sphere complexes with the binding sites on the adsorbent.^33^ Even though SO_4_^2−^ is a multivalent anion possessing a similar structure as HCrO_4_^−^ and can compete with Cr(vi), and form both inner and outer sphere complexes,^33,51^ it had a small effect on the removal of Cr(vi). This suggests that the high selectivity of the prepared PANI/Ag (AMPSA)/GO QDs NC for Cr(vi) may be due to its ability to reduce Cr(vi) ions to Cr(iii).^52,53^ Because of the partial reduction of Cr(vi) to Cr(iii) by the PANI/Ag (AMPSA)/GO QDs NC, the equilibrium (eqn (5)) is shifted in the forward direction, which promotes the further adsorption and reduction of Cr(vi).

It was also found that presence of 60 mg L^−1^ of Cr(vi) with multi ions did not significantly affect the removal percentage by PANI/Ag (AMPSA)/GO QDs NC.

#### 3.1.8 The removal of Cr(vi) using the prepared adsorbents

To highlight the superiority of the PANI/Ag (AMPSA)/GO QDs NC, the *R* and *q* of Cr(vi) ions from aqueous solutions using different adsorbents were compared, including GO QDs, Ag (AMPSA) NPs, PANI, PANI/Ag (AMPSA) NC, PANI/GO QDs NC and PANI/Ag (AMPSA)/GO QDs NC, as shown in Table 3. A dose of 1 g L^−1^ of each adsorbent was used to treat 25 mL solution of 60 mg L^−1^ Cr(vi) at pH 2 for 60 min at 30 °C.

**Table 3:** *R*% and *q* of Cr(vi) solution (60 mg L^−1^) using different adsorbents at pH 2 after 60 min at 30 °C

Adsorbent (1 g L^−1^)	*R* (%)	*q* (mg g^−1^)
GO QDs	77.7	46.64
Ag (AMPSA) NPs	70.6	42.37
PANI	81.4	48.84
PANI/Ag (AMPSA)	83.9	50.39
PANI/GO QDs	84.2	50.49
PANI/Ag (AMPSA)/GO QDs NC	99.9	59.96

Pure GO QDs exhibit small values of *R*% and *q* of 77.7% and 46.64 mg g^−1^, respectively, for the adsorption of Cr(vi) through electrostatic interactions coupled with Cr(vi) reduction to Cr(iii) and followed by Cr(iii) complexation.^48,54^ At a low pH value of 2, the removal of Cr(vi) in the aqueous phase is faster since the negatively charged Cr(vi) species migrate to the positive surface of the GO QDs (protonated hydroxyl groups and carbonyl groups) with the help of electrostatic driving forces. Cr(vi) reduction to Cr(iii) by contact with the electron–donor groups of GO QDs (OH^−^ groups) occurs during the adsorption process and the reduced Cr(iii) remains in the aqueous solution.^48^ However, the low Cr(vi) *R*% and *q* of GO QDs are attributed to the aggregation resulting from the strong van der Waals interactions and π–π stacking of the graphene oxide, which reduce the availability of effective sorption area, hence decreasing the overall metal removal and capacity.^55^

Ag (AMPSA) NPs produced small values of Cr(vi) *R*% and *q* of 70.6% and 42.37 mg g^−1^, respectively at pH 2. The removal of Cr(vi) using Ag (AMPSA) NPs is mainly related to the amide (–CONH) and sulfonyl (–SO_3_^−^) groups on the structure of AMPSA-capped Ag NPs.^56^ The combination of Cr(vi) reduction and chelation of Cr(iii) by the electron donating nitrogen of the (–CONH) groups and the ion exchange between (–SO_3_^−^) and the HCrO_4_^−^ ions is attributed for the removal of Cr(vi) by Ag (AMPSA) NPs. However, the small values of Cr(vi) *R*% and *q* are due to the aggregation of the Ag (AMPSA) NPs, which significantly decreases their high capacity.^18^

PANI presented moderate values of Cr(vi) *R*% and *q* of 81.4% and 48.84 mg g^−1^, respectively at pH 2. The enhancement in the Cr(vi) *R* and *q* values is due to the ion exchange between the dopant (–SO_3_^−^) and the HCrO_4_^−^ ions as well as the surface adsorption and reduction by the electron donating groups on the polyaniline structure.

As expected, the PANI/Ag (AMPSA) and PANI/GO QDs nanocomposites exhibited Cr(vi) *R*% values of 83.9% and 84.2%, and *q* values of 50.49 and 50.39 mg g^−1^, respectively. These results are higher than that obtained for pure PANI due to the presence of the Ag NPs and GO QDs, which increase the adsorption surface area of the nanocomposites and consequently enhance the interaction with the Cr(vi) ions.^57^ The superior removal percentage and capacity of Cr(vi) by PANI/Ag (AMPSA)/GO QDs (99.9%, 59.96 mg g^−1^) among the adsorbents are attributed to the synergistic effect of PANI, Ag (AMPSA) NPs and GO QDs, which increases the accessible binding sites on the adsorbent surface.^58^

The adsorption capacity of PANI/Ag (AMPSA)/GO QDs NC was compared to that of other adsorbents reported in previous literature, as presented in Table 4.

**Table 4:** Comparison of the Cr(vi) adsorption capacity of PANI/Ag (AMPSA)/GO QDs with that of other composites

Adsorbent	pH	*q*_m_ (mg g^−1^)	Reference
PANI/sawdust/polyethylene glycol	2.0	3.20	59
Reduced graphene oxide-montmorillonite nanocomposite	2.0	12.86	60
PANI/polygorskite	5.5	16.45	61
PANI/akaganéite	2.2	17.36	19
Activated carbon/micro-sized goethite	5.6	27.20	62
PANI/humic acid	5.0	29.26	63
Polypyrrole/titanium(iv) phosphate	2.0	31.64	64
PANI/multiwalled carbon nanotubes	4.5	31.75	65
PANI-coated ethyl celluloses	1.0	38.76	66
Kapok fiber/PANI	4.5	44.05	67
PANI/magnetite	7.6	54.00	68
PANI/multiwalled carbon nanotubes	2.0	55.55	69
Hierarchical PANI–polystyrene	4.0	58.00	70
PANI/Ag (AMPSA)/GO QDs	2.0	59.96	Present study

#### 3.1.9 The removal of Cr(vi) from water samples

For real-time application in the purification of water, two water samples containing 60 mg L^−1^ Cr(vi) ions were prepared from tap water and raw water from a drinking water canal (Alexandria, Egypt) for comparison. The dosage of PANI/Ag (AMPSA)/GO QDs NC was 1 g L^−1^ and the results are listed in Table 5. After adsorption, more than 98% of the Cr(vi) ions was removed from the above water samples. The results demonstrate that the PANI/Ag (AMPSA)/GO QDs NC exhibits potential for the removal of Cr(vi) from water samples.

**Table 5:** The *R*% of Cr(vi) ions from two water samples using the PANI/Ag (AMPSA)/GO QDs NC^a^

Sample	Cr(vi) (mg L^−1^)	Cr(vi) spiked (mg L^−1^)	Cr(vi) found (mg L^−1^)	*R*%
Tap water	N.D.	60 mg L^−1^	0.1172	99.8
Raw water	N.D.	60 mg L^−1^	0.9323	98.4

a N.D.: not detected.

#### 3.1.10 Regeneration and reusage of PANI/Ag (AMPSA)/GO QDs NC

For large-scale application, the ability to reuse an adsorbent is a crucial parameter for the decontaminating process from an economic point of view. Therefore, regeneration studies were carried out to determine if the PANI/Ag (AMPSA)/GO QDs NC can be reused after three consecutive adsorption–desorption cycles for the removal of 60 mg L^−1^ of Cr(vi) from aqueous solutions. The results are shown in Table 6. The results show that the PANI/Ag (AMPSA)/GO QDs NC could be used for three treatment cycles without loss in its adsorption efficiency. This high Cr(vi) removal efficiency of the regenerated adsorbent with an increase in recycle time may be ascribed to the presence of different active sites within the PANI/Ag (AMPSA)/GO QDs NC, which can be regenerated effectively using an eluent solution. However, Table 6 shows that Cr(vi) desorption efficiency was very low, confirming that the adsorption of Cr(vi) by PANI/Ag (AMPSA)/GO QDs NC is chemical adsorption (irreversible) such as complexation and reduction. The desorption (%) increased with the cycle number, which suggests that the capacity of the PANI/Ag (AMPSA)/GO QDs NC to reduce Cr(vi) to Cr(iii) decreased after each cycle.^32^

**Table 6:** Three consecutive adsorption–desorption cycles of PANI/Ag (AMPSA)/GO QDs NC for the removal of Cr(vi) (60 mg L^−1^) at pH 2 after 60 min at 30 °C

Number of cycles	Cr(vi) adsorption%	Cr(vi) desorption%
1	99.9	0.6
2	99.8	0.9
3	99.7	1.4

## 4. Conclusions

In this work, a ternary polymer nanocomposite-based adsorbent, PANI/Ag (AMPSA)/GO QDs NC, was prepared for the removal of Cr(vi) from aqueous solution. The adsorbent was synthesized *via* the *in situ* chemical oxidative polymerization of aniline monomers in the presence of Ag (AMPSA) NPs and GO QDs. The results indicated that the removal efficiency of the PANI/Ag (AMPSA)/GO QDs NC was highly pH dependent, and 99.9% removal and 59.96 mg g^−1^ maximum adsorption capacity were obtained at pH 2.0, 60 mg L^−1^ of Cr(vi) and 1 g L^−1^ NC dose after 60 min. The EDX and ICP studies suggested that the removal of Cr(vi) by the PANI/Ag (AMPSA)/GO QDs NC occurred through ion exchange with the dopant –SO_3_^−^ ions in the structure of the adsorbent and the subsequent reduction of Cr(vi) to Cr(iii) by the PANI and GO QDs. The PANI/Ag (AMPSA)/GO QDs NC showed high selectivity for Cr(vi) in aqueous solution in the presence of other anions and cations. Cr(vi) adsorption by PANI/Ag (AMPSA)/GO QDs NC followed pseudo-second order kinetics. The activation energy for the adsorption process was estimated to be 32.4 kJ mol^−1^, suggesting the chemisorption of Cr(vi) by the PANI/Ag (AMPSA)/GO QDs NC. The PANI/Ag (AMPSA)/GO QDs NC could be used for three treatment cycles without any loss in its adsorption efficiency. Therefore, considering its exceptional adsorption of Cr(vi) ions, good biocompatible properties and ability for reuse, the PANI/Ag (AMPSA)/GO QDs NC will be an ideal cost-effective candidate in the environmental processing field.

## Conflicts of interest

There are no conflicts to declare.

## Supplementary Material
